# Separation, reserve estimation and radioactivity responsibility of the economic heavy minerals of East El- Arish black sand, North Sinai, Egypt

**DOI:** 10.1038/s41598-023-31440-y

**Published:** 2023-03-21

**Authors:** M. Awad, A. M. El Mezayen, S. M. El Alfi, H. H. Ali, M. I. Sayyed, M. Y. Hanfi

**Affiliations:** 1 Nuclear Materials Authority, El Maadi, PO Box 530, Cairo, Egypt; 2 Department of Geology, Faculty of Science, Al Azhar University Nasr City, Cairo, Egypt; 3 Department of Physics, Faculty of Science, Isra University, Amman, Jordan; 4 Department of Nuclear Medicine Research, Institute for Research and Medical Consultations (IRMC), Imam Abdulrahman Bin Faisal University (IAU), PO Box 1982, Dammam, 31441 Saudi Arabia; 5 Ural Federal University, Mira Street 19, Ekaterinburg, Russia 620002

**Keywords:** Solid Earth sciences, Geology, Mineralogy, Sedimentology

## Abstract

Multi-heavy mineral separation techniques like density, magnetic, and electromagnetic techniques are followed by picking, grain counting, and mineralogical examinations conducted with an environmental scanning electron microscope (ESEM). These techniques have been applied to several drill hole (well) composite samples representing beach sand and dunes of East El-Arish area, North Sinai, Egypt. The results explain the remarkable enrichment in total heavy minerals (THM) of beach sand (4.4%) compared to sand dunes (2.6%): this is due to the natural concentration of the currents of the Mediterranean Sea. After characterizing the heavy minerals in terms of the abundance of each mineral species, the mean values of content relative to total economic heavy minerals (TEHM) were determined as 70.03% ilmenite, 3.81% leucoxene, 3.03% magnetite, 8.7% garnet, 10.6% zircon, 3.13% rutile, 0.37% monazite, and 0.31% titanite in the sand dune samples. The total economic heavy minerals in the beach samples were 64.08% ilmenite, 1.6% leucoxene, 2.84% magnetite, 18.16% garnet, 10.4% zircon, 2.18% rutile, 0.61% monazite, and 0.13% titanite. Monazite, thorite, zircon, and apatite are the main radioactive minerals in the study area. The combination of two specific characteristics of the studied monazite (relatively high Th-U content and high frequency) makes monazite the main contributor to radioactivity of the study area.

## Introduction

Economic heavy mineral deposits are detritus sediments that accumulate in coastal areas, including beach sand and sand dunes. These deposits are derived from the weathering of metamorphic and igneous rocks. Rivers and aeolian processes transport sediments to coastal areas, where they are sorted and concentrated by water, tidal, and wind currents. These processes lead to the accumulation of layers of dense sediments in a variety of coastal depositional environments in the form of beach sand and sand dunes^1,2^. These deposits contain strategic and economic heavy minerals needed for the nuclear industry and other metallurgical and engineering industries. Worldwide studies of heavy minerals have focused on the economic importance of these deposits, including in the United States^2^, Brazil^3^, Australia^4,5^, India^6^, and Egypt^7–16^. These studies show that the main heavy minerals are ilmenite, magnetite, garnet, rutile, zircon, titanite, apatite, monazite, and others.

In recent years, there has been growing interest in the extraction of economically valuable heavy minerals from Egyptian black sand located on the country’s northern coast, especially after the foundation of the Egyptian Black Sand Company (EBSC) in 2015 in collaboration with the Egyptian Nuclear Materials Authority (NMA). The latter has formulated an industrial plan for the exploitation of black sand sediments in the coastal area of the Mediterranean from Roseta in the west to Rafah in the east, including north Sinai sand dunes and beach sand. To continue and complete this plan, the evaluation and study of the economic heavy minerals in north Sinai are required to supply and support the production line. After providing a literature survey of work performed in areas like Roseta, Damietta, and the El-Arish coast, the present work investigates the mineralogy of economic heavy minerals, including radioactive minerals like monazite, zircon, and thorite, in sand dunes and beach sediments. Consequently, the paper discusses the relationship between radioactivity and the distribution of heavy minerals in the studied samples.

**Figure 1:**
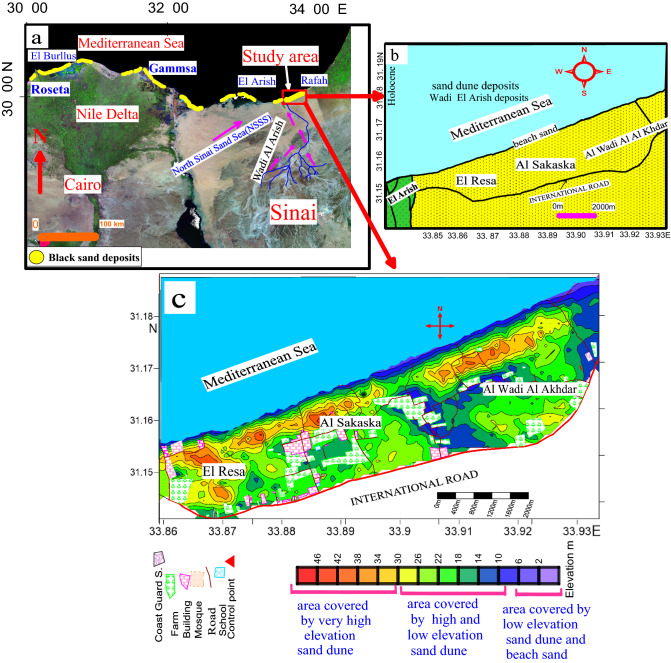
(**a**) Noth Egypt satalight image shows location of the main black sand deposits and the study area. (**b**) Geological map of the study area.(**c**) Detailed topographic map illustrate the main deposits for study area (the area only covered by sand dune and beach sand), Map was created by Surfer Software 13.6 (2016) https://www.goldensoftware.com/products/surfer.

The study area is located in the northern part of Sinai between latitudes 31° 11′ 05.4"–31° 08′ 31.7" N and longitudes 33° 57′ 45.2"–33° 51′ 40.3" E (Fig. 1). The map provided by El Hadary^11^ shows 13 main black sand localities on Egypt’s Mediterranean coast. These localities are plotted in the following satellite image of northern Egypt (Fig. 1a). The study area covers about 26 km^2^. It is part of the North Sinai Sand Sea (NSSS). This extends over ~ 120 km from the Suez Canal in the west to the eastern borders of Egypt and 30–120 km from the southern slopes of Gabal Maghara and Gabal Halal in the south to the Mediterranean coast in the north^17^. According to Roskin^18^, the NSSS covers ~ 13,600 km^2^. Several contributions have been made to describing the geomorphology of NSSS^17–19^. According to the provided geological map (Fig. 1b), Holocene sand dunes and beach sand cover the study area. A description of the main geomorphologic units is provided the next section.

The geological source of the sand dunes in the NSSS is mostly exogenous, because, although there is a tremendous amount of sand in this area, there are no significant surface sandstone formations. In addition, the biggest fluvial system, i.e., Wadi Al-Arish, which originates from the middle Sinai and flows northward to the Mediterranean (Fig. 1a), cannot be considered as the main source of sediments for this dune field simply because this valley drains limestone strata and the sand in the NSSS is mainly quartz^19^. The possibility that this sand came from the Nile River through the defunct Pelusiac Nile branch^20^, which was located west of the sand sea, is quite convincing. The wind moved sediment from the Nile that settled along the coast to form this coastal sand sea^19^. Overall, the mineralogical data from Muhs^19^ suggest that the dunes are derived dominantly from the Nile delta, with Wadi Al Arish sands being a minor contributor.

## Geomorphology of the study area

### Coastal sand dune

**Figure 2:**
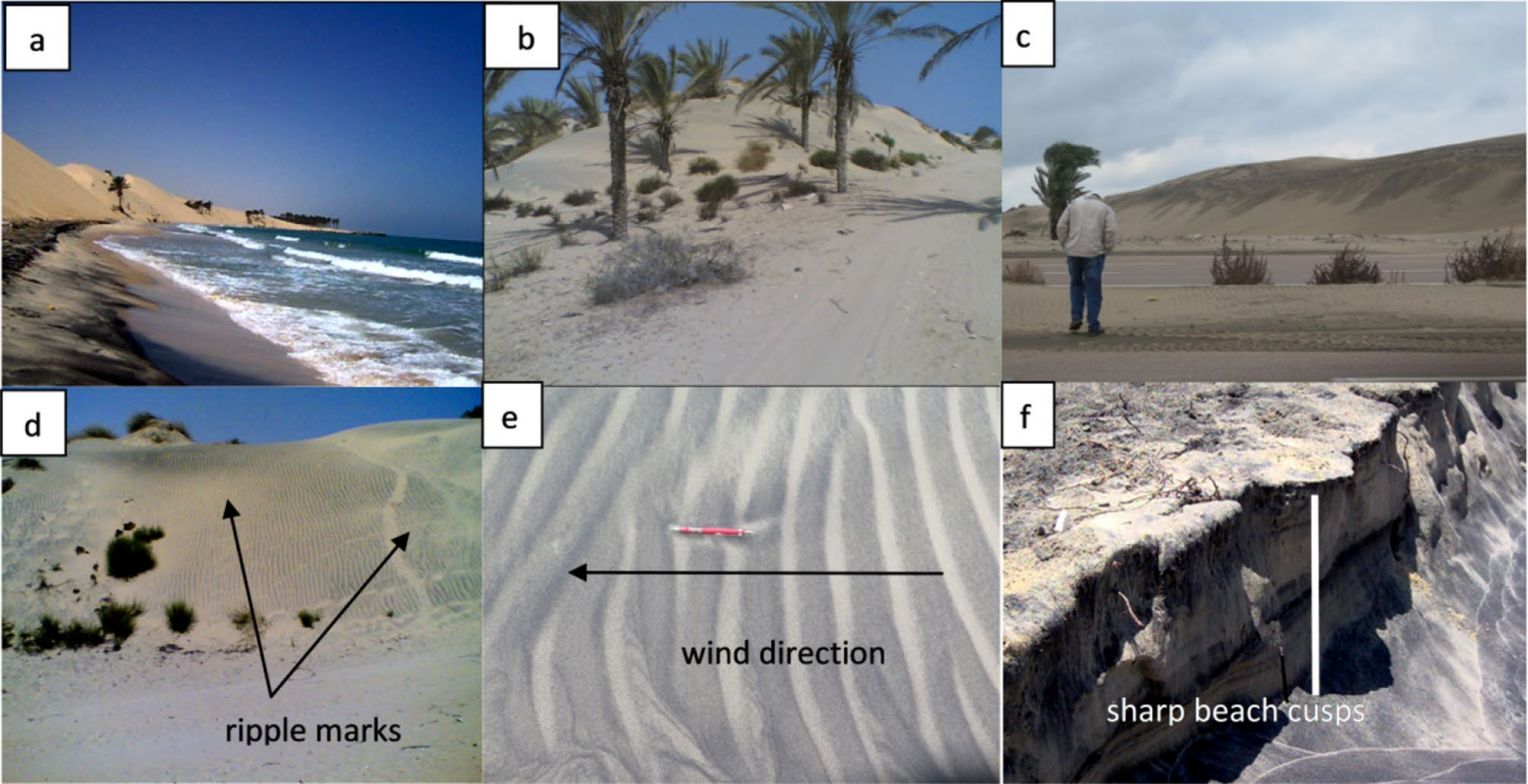
Photographs show: (**a**) linear sand dune with concave shape of coastline, (**b**) barchan sand dune (**c**) transverse dune, (**d**) sand dune with ripple marks on its limbs, (**e**) the ripple marks and (**f**) sharp beach cusps of about 75 cm height.

The study area is part of the NSSS. The common geomorphologic units in the study area are coastal sand dunes and beach sands (Fig. 1c). Coastal sand dunes generally extend almost parallel to the area’s regional structure, with elevations up to 48 m. Three types of sand dunes are observed in the study area, namely (in order of abundance): (1) linear (longitudinal), (2) barchan, and (3) transverse. *Linear dunes* are the most abundant aeolian landform on the northern Sinai coast. They extend along the coastal zone of the Mediterranean. They consist of an array of parallel to sub-parallel vegetated linear dunes (Fig. 2a). Their length ranges from a few hundred meters to more than 1 km, while their width ranges from 50 to 100 m: they generally have wavy crests. *Barchan dunes* are particularly pronounced in the El-Arish region (El Resa, Al Sakaska, Al Wadi Al Akhdar) (Fig. 2b). These dunes are 10 m thick and relatively vegetation-free^21^. These dunes are formed by southwestern winds and migrate in the NE direction, where the supply of sand is somewhat limited. *Transverse dune* belts are superimposed on older anchored linear ridges, forming complex dunes. They are generally arranged parallel to each other. The transverse ridges are generally oriented in a NNW-SSE direction (ranging from N5°W to N12°W). In the north, their slip faces may extend for about 1.9 km and have an average width of about 200 m (Fig. 2c). Ripple marks have been produced as a result of wind action on the limbs of the sand dunes (Fig. 2d,e).

### Beach sand

The Holocene has seen great changes in the position of shorelines and coastal morphology as a result of rising sea levels^22^. From west to east, the prevailing Mediterranean current reshapes coastlines. The Mediterranean current plays a vital role in physically concentrating heavy minerals along beach lines. Sinai beaches consist of unconsolidated and readily transportable sand; they adjust quickly to changes in the nature and energy of coastal processes. Large asymmetric cusps have been identified along the beach in the study area (Fig. 2f).

## Materials and method

### Sampling and techniques

**Figure 3:**
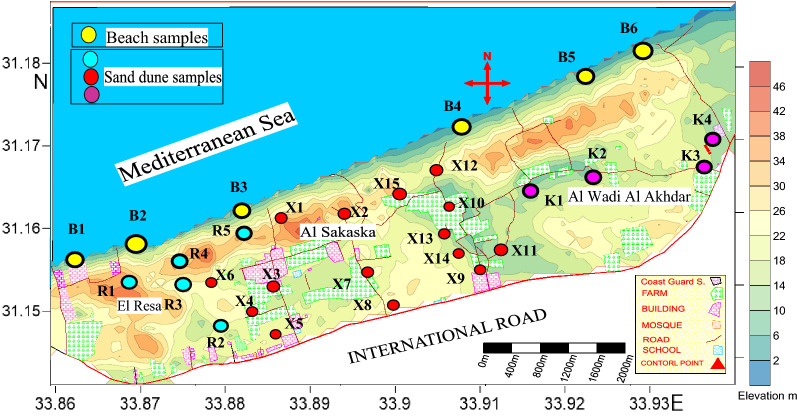
Topographic map shows samples location. Map was created by Surfer Software 13.6(2016) .https://www.goldensoftware.com/products/surfer.

30 drill hole (well) samples representing the main two geomorphologic units in the studied area (beach sand and sand dunes) were homogeneously mixed and collected to give one composite sample for each well. The depth of the wells varied from 10 to 35 m, with a mean of 25 m for the sand dunes and 6 m for the beach sand. The collected samples are distributed as follows: 5 samples from the El Resa area (labelled as R_n_), 15 samples from the Al Sakaska area (labelled as X_n_), 4 samples from the Al Wadi Al Akhdar area (labelled as K_n_), and 6 samples from the beach (labelled as B_n_) (Fig. 3).

All mineral species with a specific density more than 2.88 g/cm^3^ are considered heavy minerals. The total heavy minerals are concentrated in the medium, fine, and very fine sand-size categories^23^. These investigations were conducted on sand less than 0.5 mm in size. The original sand samples were dried and prepared for mineral separation using bromoform (Sp. Gr. 2.88 g/cm^3^). The heavy groups were dried and weighed. Their compositions are calculated and provided in Table 1. The light groups were discarded.

**Table 1:** Percentages of total heavy minerals (THM) separated by bromoform solution for the studied sediments.

Unit	Area	S. No	THM %	Unit	Area	S. No	THM %
Coastal sand dune	El Resa	R1	2.27	Beach sand	Beach sand	B1	2.69
		R2	2.00			B2	3.13
		R3	2.82			B3	2.08
		R4	2.87			B4	11.39
		R5	2.02			B5	5.19
	Al Sakaska	X1	2.10			B6	3.02
		X2	2.50			Min	2.08
		X3	3.01			Max	11.39
		X4	2.30			Ave	4.583
		X5	2.40				
		X6	2.20				
		X7	2.44				
		X8	2.64				
		X9	3.73				
		X10	2.78				
		X11	2.87				
		X12	3.14				
		X13	3.20				
		X14	2.67				
		X15	2.81				
	Al Wadi Al Akhdar	K1	2.35				
		K2	2.98				
		K3	2.37				
		K4	2.08				
		Min	2.00				
		Max	3.73				
		Ave	2.61				

Magnetite separation was carried out using a small hand magnet of a suitable strength. The magnetite-free heavy minerals were magnetically fractionated using a Frantz Isodynamic Magnetic Separator (Model L-1) with a side slope of 5° and a forward slope of 20°^24^.

The results of magnetic separation were examined with a binocular stereo microscope to determine the proportions of minerals in each fraction. Counting was done for a small representative sample (about 0.1 g) of each fraction and sprinkled as a one-grain-thick layer on a glass plate. The counting of each mineral was carefully executed, and the frequency was calculated according to the following equation^13,25^:

$${\text{Wt }}\% \, = \frac{N \times d}{T} \times {1}00$$

N: number of grains for each mineral;d: specific density of each mineral;T: sum. of numbers of grains for each mineral multiplied by its density (i.e., T = ∑(N_1_*d_1_) + (N_2_*d_2_) + ………….. (N_x_*d_x_), where 1, 2, and x refer to the prepared mineral fractions).

The frequencies of each mineral in the different magnetic groups are recorded in Table 2.

**Table 2:** The percentages of heavy minerals for studied sediments.

Area	TEHM									∑TEHM%	GS %	THM %
	Opaque Minerals %					Non-opaque minerals %						
	S.No	Mgt.%	Ilm.%	Leuc.%	Rut.%	Zr.%	Gar.%	Mz.%	Tit.%			
Coastal sand dune												
El Resa	R1	0.031	1.133	0.063	0.090	0.095	0.177	0.004	0.005	1.597	0.668	2.265
	R2	0.046	1.065	0.032	0.048	0.134	0.113	0.047	0.008	1.492	0.508	2.000
	R3	0.052	1.410	0.049	0.033	0.284	0.161	0.010	0.000	1.998	0.821	2.819
	R4	0.056	1.365	0.082	0.071	0.238	0.186	0.012	0.009	2.019	0.851	2.870
	R5	0.046	1.167	0.037	0.060	0.138	0.109	0.004	0.004	1.564	0.456	2.020
Al Sakaska	X1	0.052	1.367	0.039	0.036	0.181	0.070	0.001	0.003	1.749	0.351	2.100
	X2	0.038	1.262	0.129	0.102	0.183	0.294	0.004	0.026	2.038	0.463	2.500
	X3	0.044	1.372	0.067	0.048	0.273	0.257	0.004	0.001	2.066	0.944	3.010
	X4	0.026	1.208	0.027	0.059	0.176	0.166	0.001	0.000	1.662	0.636	2.298
	X5	0.091	1.171	0.075	0.082	0.244	0.142	0.006	0.005	1.817	0.583	2.400
	X5	0.035	1.060	0.057	0.039	0.140	0.079	0.008	0.006	1.424	0.776	2.200
	X7	0.034	1.287	0.053	0.047	0.164	0.258	0.001	0.004	1.848	0.592	2.440
	X8	0.043	1.057	0.126	0.042	0.185	0.157	0.005	0.013	1.628	1.012	2.640
	X9	0.071	1.451	0.145	0.056	0.387	0.237	0.009	0.009	2.365	1.366	3.730
	X10	0.119	1.457	0.077	0.066	0.349	0.141	0.007	0.007	2.224	0.556	2.780
	X11	0.072	1.300	0.076	0.049	0.237	0.158	0.001	0.011	1.904	0.966	2.870
	X12	0.152	1.753	0.121	0.057	0.175	0.208	0.013	0.008	2.487	0.653	3.140
	X13	0.100	1.880	0.067	0.079	0.241	0.237	0.002	0.007	2.612	0.588	3.200
	X14	0.039	1.373	0.096	0.054	0.181	0.109	0.003	0.010	1.865	0.805	2.670
	X15	0.071	1.500	0.071	0.069	0.178	0.154	0.002	0.005	2.049	0.757	2.806
W Akh	K1	0.038	1.424	0.035	0.057	0.161	0.199	0.001	0.005	1.921	0.429	2.350
	K2	0.067	1.797	0.077	0.090	0.230	0.118	0.009	0.003	2.390	0.590	2.980
	K3	0.038	1.197	0.078	0.038	0.170	0.167	0.006	0.001	1.696	0.673	2.370
	K4	0.032	1.117	0.063	0.075	0.140	0.112	0.000	0.001	1.540	0.540	2.080
	Min	0.026	1.057	0.027	0.033	0.095	0.070	0.000	0.000	1.424	0.351	2.000
	Max	0.152	1.880	0.145	0.102	0.387	0.294	0.047	0.026	2.612	1.366	3.730
	Aveg	0.058	1.341	0.073	0.060	0.203	0.167	0.007	0.006	1.915	0.691	2.606
Beach												
Beach	B1	0.091	1.176	0.082	0.221	0.072	0.048	0.006	0.007	1.703	0.991	2.694
	B2	0.072	1.828	0.094	0.045	0.275	0.315	0.019	0.003	2.650	0.476	3.126
	B3	0.052	1.258	0.032	0.032	0.231	0.156	0.006	0.002	1.767	0.317	2.084
	B4	0.309	7.065	0.095	0.116	1.238	1.998	0.066	0.003	10.891	0.504	11.394
	B5	0.125	2.553	0.063	0.075	0.426	0.984	0.029	0.016	4.272	0.920	5.191
	B6	0.026	1.277	0.010	0.028	0.220	0.798	0.019	0.001	2.379	0.639	3.018
	Min	0.026	1.176	0.010	0.028	0.072	0.048	0.006	0.001	1.703	0.317	2.084
	Max	0.309	7.065	0.095	0.221	1.238	1.998	0.066	0.016	10.891	0.991	11.394
	Aveg	0.112	2.526	0.063	0.086	0.410	0.716	0.024	0.005	3.943	0.641	4.584

S. No. = Sample number, Mgt. = Magnetite, Ilm. = Ilmenite, Leuc. = Leucoxene Rut. = Rutile Zr. = Zircon, Gar. = Garnet, Mz. = Monazite, Tit. = Titanite, GS = Green silicates, TEHM = Total Economic Heavy Minerals, THM = total heavy minerals; W Akh = Al Wadi Al Akhdar.

Mineralogical examinations were performed with a Phillips XL-30 environmental scanning electron microscope (ESEM), supported by energy-dispersive X-ray spectroscopy (EDX) and observations of heavy minerals under a binocular stereo microscope. This illustrated grain morphologic characteristics such as crystal habit, color, surface pitting, rounding, and abrasion. Radiometric measurements of eU, eTh, and K were carried out using a Bicron scintillation NaI (Tl) detector connected to a multichannel analyzer.

## Results and discussion

**Figure 4:**
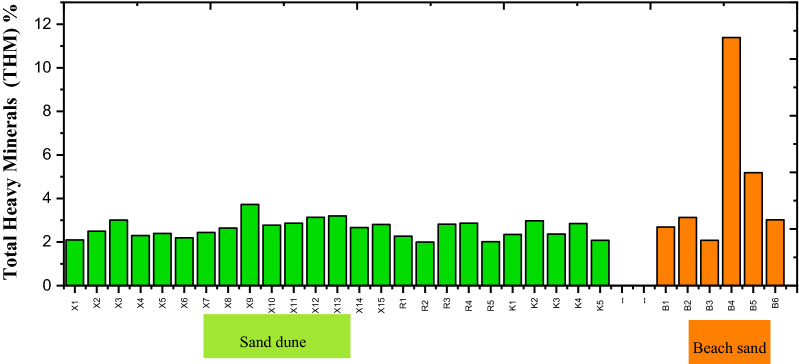
The histogram shows the total heavy minerals distribution among the sand dune and beach sand.

**Figure 5:**
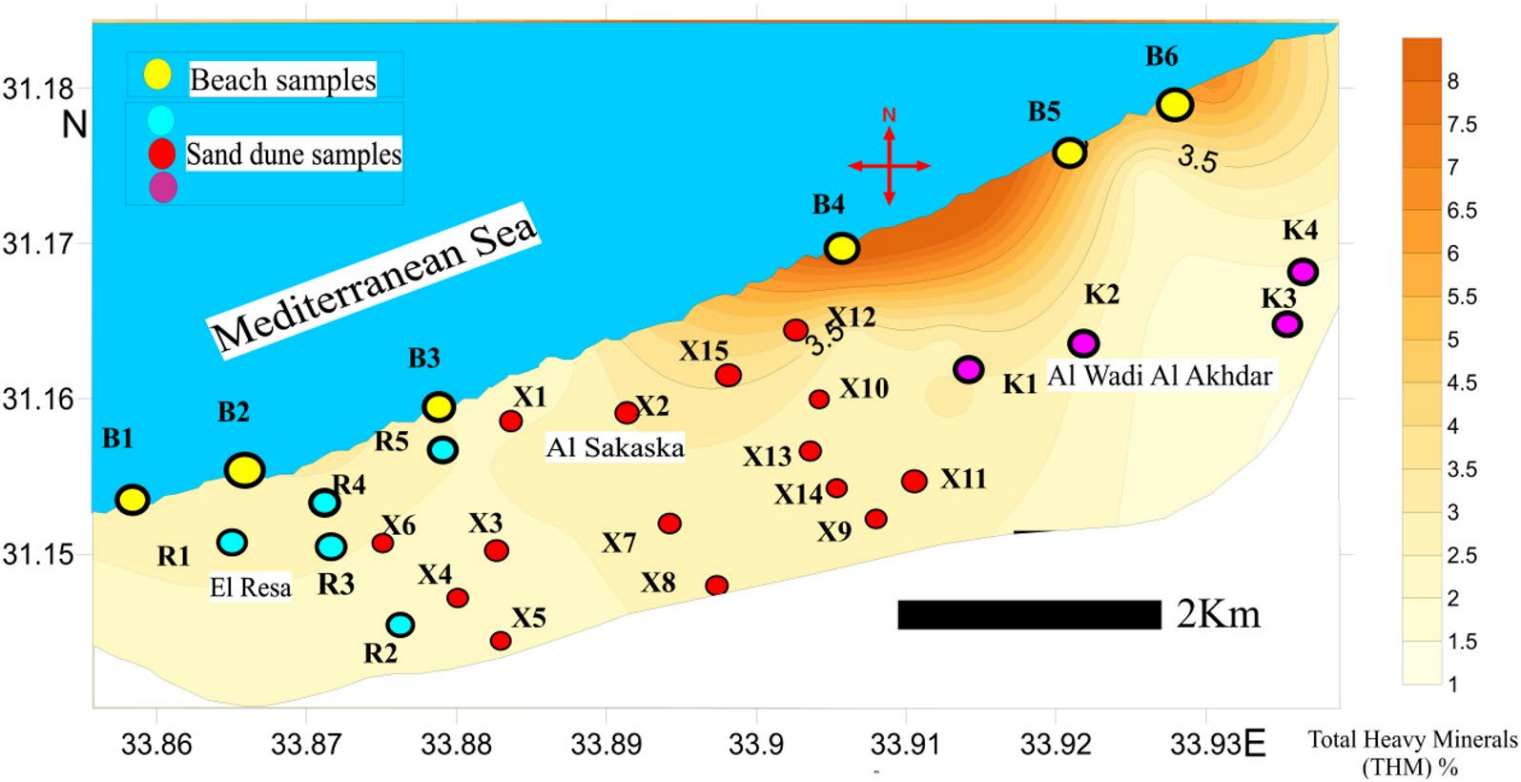
Contour map shows the total heavy minerals distribution. Map was created by Surfer Software 13.6 (2016). https://www.goldensoftware.com/products/surfer.

**Figure 6:**
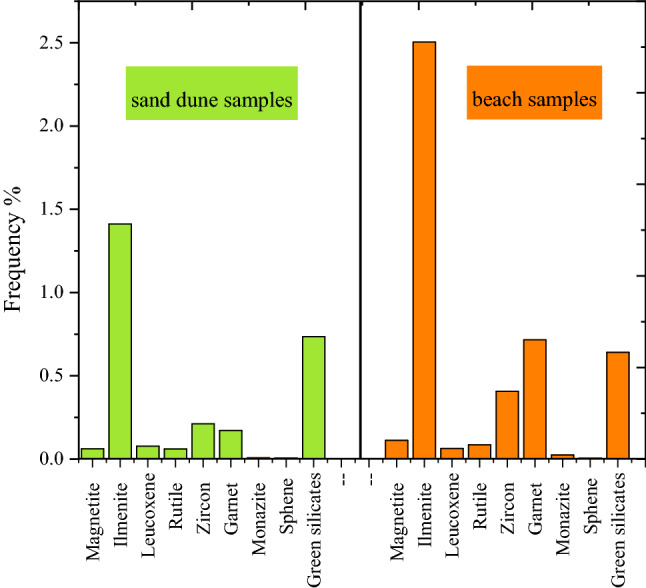
Frequency % of each individual heavy mineral among the study area.

**Figure 7:**
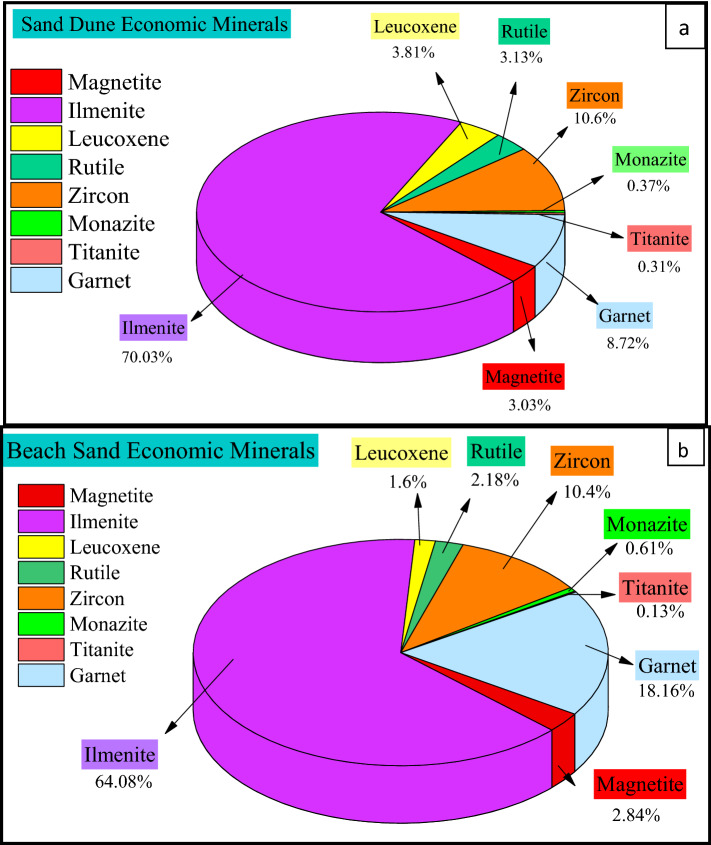
Pie diagram illustrates the frequency % of each individual heavy mineral relative to total economic heavy minerals (TEHM) for (**a**) sand dune and (**b**) beach sand.

The study of heavy minerals is focused on those of high economic value, namely total economic heavy minerals (TEHM). Those with the lowest economic value, namely green silicates (amphiboles, pyroxene, mica, and others), are ignored. The distribution of the total heavy minerals is graphically represented with both a histogram (Fig. 4) and a contour map (Fig. 5). The histogram reveals the remarkable enrichment of total heavy minerals in the beach samples compared to the sand dune samples. In addition, the contour map indicates the presence of two agglomerates of heavy minerals located near the beach in the Al Wadi Al Akhdar area (Fig. 6). The beach sand, in general, and the two agglomerates, in particular (samples B4 & B5), are characterized by high THM content, high monazite content, and high eTh: see the contour map (Fig. 7) in the next section. Therefore, these two agglomerates are responsible for radioactivity in the area. However, the comparison between the important parameters of the Egyptian black sand localities were tabulated in table 3.

**Table 3:** comparison between the important parameters of various Egyptian black sand localities.

Area	El Arish (Present study)	El Burullus and Roseta
Elevation of sand dune	High elevation (about 5- 48 m)	Low elevation( 5- 25 m);^9,13^
Grain size	Fine to very fine	medium to fine^11,13^
Type of dune	Longtudinal type is the most dominant^19^	barchan type is the most dominant
Geological source of dune	there are two sources 1- Nile delta through anceient branches of Nile River 2- Wadi El-Arish as minor contributor^19^	One source; Nile River^9,11^
Radioactivity		
uranium (eU) ppm	0.5 to 4 ppm	3 to 7 ppm^9,26,27^
Thorium (eTh)ppm	0.5 to 14 ppm	8 to 25 ppm;^9,26^
Total heavy minerals(THM)	2.6% for sand dune 4.4% for beach sand	16.6% east Roseta area;^9^ 8.7% Dameitta area^12^ 3.5%West El Burullus area^12^

### Opaque minerals

#### Magnetite Fe^2^^+^Fe^3^^+^_2_O_4_

Magnetite was separated using a hand magnet. It was recorded in all studied sediments: its content in the coastal dune samples ranges from 0.026 to 0.152%, with an average of 0.058%, and ranges from 0.026 to 0.309%, with an average of 0.112%, in the beach samples (Table 2 and Fig. 6). The magnetite is massive, granular, and angular to sub-angular (Fig. S1a): octahedron crystals of magnetite are less frequent. The magnetite occurs also as isolated grains or as parallel twins and irregular shapes (Fig. S1c). The selected magnetite grains were investigated using ESEM (Fig. S1b, c and d). Rounded magnetite grains are shown in Fig. S1b. In the present study, EDX microanalysis data shows that the additional weight percentage of titanium reaches up to 28.64% (Fig. S1d). Magnetite has different amounts of trace elements (e.g., Mg, Al, Sc, Ti, V, Nb, Cr, Ge, Co, Ni, Zn, Mn, Ga, Y, Ta, and Zr) in its crystal structure during formation processes^28,29^. The studied magnetite grains have Ti, Al, Si, and Ca as trace elements (Fig. S1). Ti–rich magnetite is related to mafic and ultramafic igneous rocks, while Ti-poor magnetite crystallized from felsic to intermediate magmas^30^. Summing up, it can be concluded that the studied magnetite high in Ti (Fig. S1d) is derived from mafic and ultramafic igneous rocks. The results are compatible with El-Kammar^8^, who stated that titano-magnetite minerals, such as magnetite, ilmenite, leucoxene, and rutile, are mainly derived from mafic volcanic rocks of Blue Nile provenance.

#### Ilmenite (FeTiO_3_)

Ilmenite is considered the most abundant heavy mineral in Egyptian black sand. Ilmenite is a ferromagnetic mineral separated mainly at the magnetic fields 0.2A and 0.5A. Morphologically, the investigated ilmenite was recorded as black with a metallic luster. It occurs as irregular to subangular shapes with smooth edges (Fig. S2a), rod-like shapes (Fig. S2b), ovals with smooth and pitted surfaces, and well-rounded to subrounded shapes (Fig. S2c) (Table 3).

The ESEM data of ilmenite are shown in Fig. S2d–g. Ilmenite contains TiO_2_ ranging from 57.18 to 59.94%; this agrees with Garnar^31^, who states that TiO_2_ in ilmenite ranges from 45 to 65%. In addition, several studies have noted the presence of Ti oxide in ilmenite (Table 4). El-Arish ilmenite is considered high grade due to the relatively high content of TiO_2_ (57–60%) compared to other Egyptian detrital ilmenites (Table 4). MnO % was used as a source rock indicator:^30,32^ analysis shows that ilmenite grains with MnO > 2.0 wt% are prevalent in sands from felsic igneous rocks. Some grains of detrital ilmenite are measured here (Fig. S2f.) with MnO > 2.0 wt%, suggesting they originate in felsic igneous rocks. Ilmenite represents about 70% and 64% of TVHM for sand dunes and beach sand, respectively (Fig. 7). Ilmenite content ranges from 1.057 to 1.880%, with an average of 1.341%, in the coastal dune samples and from 1.176 to 7.065%, with an average of 2.526%, in beach sand samples (Fig. 6). Consequently, the ilmenite concentration is similar to that in the Baltim area (~ 2.07%)^9^. The EDX microanalysis of ilmenite demonstrates that ilmenite does not play a significant role in radioactivity in the study area.

**Table 4:** TiO_2_% in ilmenite of different localities and provenances.

Locallity	TiO_2_%	Provenance	References
Egypt (East El-Arish)	57–59	Coast sediments	The present study
Egypt (East Roseta)	47	Sand dunes	^16^
Egypt (Roseta)	49	Sand dunes	^7^
India	60–66	Beach sand	^33^
India	52–54	Coast sediments sediments sediments	^34,35^
Bangladesh	49–51	Sand deposits	^36^

#### Leucoxene (altered ilmenite FeTiO_3_)

Leucoxene grains were separated at 0.5A and 1.0A, although the majority were separated at 1.0A. Leucoxene was recorded in all studied sediments, ranging from 0.027% to 0.145%, with an average of 0.073%, in the coastal dune samples and from 0.010% to 0.095%, with an average of 0.063%, in the beach samples. According to the degree of alteration, the leucoxene’s color ranges from black (Fig. S3a) to brown (Fig. S10b) and yellow (Fig. S3c). Its grains range from irregularly rounded to subrounded, platy, and prismatic with smooth or pitted surfaces. Ilmenite occurs as relicts on the surface of leucoxene, indicating the presence of alteration processes (Fig. S3d). Leucoxene grains were investigated using ESEM, and the EDX microanalysis data is shown in Fig. S3e. Leucoxene contains 87.33% TiO_2_; these results agree with Nadoll et al.^28^ who state that TiO2 in leucoxene ranges from 68 to 92%.

### Non-opaque minerals

#### Zircon (ZrSiO_4_)

Zircon is concentrated in both non-magnetic and magnetic fractions at 1.5A. Separated zircon crystals in a 1.5A nonmagnetic field have a long prismatic shape and a water-clear color with a vitreous luster^37,38^. Saxena^39^ has stated that water-clear zircon grains in all types of rocks are nonmagnetic. In ore concentration terms, the zircon content values vary from 0.095% to 0.387%, with a mean of 0.203%, in the coastal dune samples and from 0.072% to 1.238%, with a mean of 0.410%, in the beach samples. The majority of zircon shows a significant heterogeneity concerning composition^40–42^, crystal color^43^, and morphology^44,45^. Most of the zircon is colorless. According to crystal morphology, the studied zircon consists of colorless, short-to-long prismatic habits with bipyramid terminations (water clear zircon) (Fig. S4a), colorless prismatics with black inclusions (Fig. S4b), colourless oval shapes (Fig. S4c), and colourless rounded shapes (Fig. S4d). Magnetic zircon was recorded as yellow prismatics (Fig. S4e) with muddy (Fig. S4f.), orange (Fig. S4g), red, and black euhedral grains (Fig. S4h and i). The variation in the color of zircon may be attributed to the density of the fine inclusions and the degree of iron oxide staining. Some inclusions may be due to iron ions that penetrated from the original melt through the zircon lattice during magmatic crystallization or from an outside source^38^. These inclusions are considered weak points that accelerate grain disintegration.^39^.

Fig. S5 illustrates EDX microanalysis and BSE imaging of zircon crystals as follows: a) typical euhedral zircon crystals indicating a short transportation distance, b) bipyramid zircon grains, c and d) fractured prismatic zircon grains indicating uranium loss, e) irregular zircon fragments, and f) bipyramid grains. Table 5 shows that Th oxide content varies from nil to 3.96 with a mean of 2.04, while U oxide content ranges between nil and 2.50% with an average of 0.74%. Hence, this is lower than the U and Th content in thorite and monazite.

**Table 5:** EDX microanalysis data of zircon (carbon and oxygen are excluded).

Element	Colorless zircon wt%			Yellow zircon wt%	Orange zircon wt%		Red zircon wt%		Black zircon wt%
	a	b (grain1)	b (grain2)	C	d (grain1)	d (grain2)	e (grain1)	e (grain2)	f
Zr	66.37	68.82	70.62	74.99	66.63	66.19	71.87	67.46	50.48
Si	23.86	19.98	21.19	16.97	22.49	21.93	21.07	19.70	22.44
Al	1.85	0.84	1.51	1.80	0.71	1.05	1.08	0.97	2.16
U	1.08	2.55	0.00	0.00	0.00	0.00	0.56	0.00	2.50
Th	0.00	1.68	1.26	1.24	3.56	3.17	0.00	3.52	3.96
Ca	0.22	1.36	0.96	0.94	1.19	1.18	0.77	1.86	3.82
Fe	0.78	0.79	0.39	0.61	1.27	1.65	1.24	1.50	10.31
Hf	5.85	3.99	4.08	3.44	4.15	4.83	3.41	5.01	4.32
Total	100.00	100.00	100.00	100.00	100.00	100.00	100.00	100.00	100.00
Zr/Hf	11.34	17.24	17.30	21.79	16.05	13.70	21.07	13.46	11.68

The elements Zr and Hf are frequently used in sediment source discrimination^46–48^. The Zr/Hf ratio ranges 11 to 21 lower than the chondritic value of ~ 30. Furthermore, the Zr/Hf ratio generally decreases from ultramafic to felsic during simple magmatic differentiation^49^, suggesting the possible derivation of the studied zircon from more mafic rocks.

#### Garnet (Mg,Fe^2^^+^,Mn,Ca)_3_(Al,Cr,Ti,Fe^3^^+^)_2_Si_3_O_12_

Garnet is recorded in the studied samples as colorless, light pale pink irregular fragments (Fig. S6a), pinkish rounded to subrounded grains (Fig. S6b), and red and reddish-brown crystals with black inclusions (Fig. S6c). Additionally, there are other features on the grain surfaces such as surface pitting and etch facets. Figs. S7 and S8 demonstrate dissolution and transportation processes. These observations are in agreement with Mange and Maurer^50^. The EDX microanalysis of garnet grains is shown in Table 6. Based on data from Nickel and Nichols^51^, in addition to the EDX microanalysis data, the studied garnet is classified as almandine garnet (Fe-rich garnet) and an almandine-spessartine solid solution (Table 6).

**Table 6:** EDX microanalysis data of garnet.

	Alamandine garnet					Alamandine-spessartine solid solution garnet	
	A	b	C	d	e	f	g
Oxides	wt%	wt%	wt%	wt%	wt%	wt%	wt%
SiO_2_	33.98	33.20	37.82	34.41	33.25	31.35	36.35
Al_2_O_3_	20.31	19.46	19.58	20.49	19.97	17.18	21.16
Fe_2_O_3_	34.77	36.19	31.41	33.98	39.52	23.56	14.47
MgO	5.91	3.02	8.67	0.00	0.00	1.66	2.64
CaO	3.72	2.77	2.18	2.51	3.66	8.15	1.47
MnO	1.31	5.36	0.00	4.36	3.59	17.35	23.90
K_2_O	0.00	0.00	0.34	0.53	0.00	0.75	0.00

Single-grain chemistry of detrital garnet is used in sedimentary provenance analysis^52–58^. Consequently, ESEM was used to perform semi-quantitative chemical analysis for seven garnet grains to define garnet type and source rock (Table 6): this was followed using the Fe + Mn–Mg–Ca ternary plot suggested by Mange and Morton^53^. They name the garnet types A, Bi, Bii, Ci, Cii, and D type A-granulite-facies metasediments and intermediate felsic igneous rocks (high Mg, low Ca), type Bi-intermediate to felsic igneous rocks (high Fe, high Mn), type Bii-medium–low metasedimentary rocks, amphibolite-facies (low Mg, variable Ca), Ci-metabasic rocks, type Cii-ultramafic rocks, type D-low-grade metabasic rocks, and contact metasomatic metamorphic rock (Ca-rich). Such garnets are here termed “type B” (Fig. S8). It can be concluded that most garnet grains are related to Bi-type intermediate to felsic igneous rocks, while others are related to Bii-medium–low metasedimentary rocks and amphibolite-facies.

Mineral inclusions in detrital garnet were used as a provenance indicator^59^. It is concluded that metamorphic source garnet grains contain mineral inclusions ≥ 2 µm. Consequently, we recorded some grains containing such size inclusions via binocular stereo microscope observations and ESEM investigations, suggesting its derivation from a metamorphic source. Other grains have no impurities (Fig. S6a). Garnet was recorded in all studied sediments, and its content ranged from 0.070% to 0.294%, with an average of 0.167%, in the coastal dune samples and ranges from 0.048% to 1.998%, with an average of 0.716%, in the beach samples. The pie diagram (Fig. 7) illustrates the clear enrichment of garnet in beach sand (about 18% TEHM). The EDX microanalysis on garnet suggests no significant role for garnet in the natural radioactivity of the study area.

#### Monazite (Ce, La, Nd, Th, Y)PO_4_

The monazite of the studied sediments is (Ce)-Monazite. In ore concentration terms, the studied area shows a generally marked enrichment of total heavy minerals in beach sand, with an exceptional increase in THM in samples B4 and B5 (Figs. 5 and 6 and Table 2). This is due to the natural physical concentration of the Mediterranean current. The beach sand samples have more monazite mineral content (~ 0.024%) than the sand dune samples (~ 0.007%) (Table 2). So, monazite is responsible for the radioactivity of the study area. The ESEM data of monazite grains (Fig. S9) reveal that the Th oxide content varies from 10.14 to 12.67, with a mean of 11.23, while the U oxide content ranges between 1.77 and 2.67%, averaging 2.15%. The high Th content along the El-Arish beach is due to its high monazite content, containing 7.92% ThO_2_^60–62^. The mineralogical concentration data shows the enrichment of monazite over thorite; thus, the radioactivity of the studied area is related to monazite rather than thorite. The ESEM data for the monazite grains display Th and U content, indicating that radioactivity is mainly related to thorium rather than uranium. Furthermore, the mineralogical concentration data from THM, especially monazite and thorite, is compatible with the radioactivity data in the next section. Moreover, according to Fig. S9b, d, and f, monazite grains contain about 60% of REE, with remarkable dominance of the lightest four elements (La, Ce, Pr, and Nd), which make up more than 80% of the total REE. The abundance of LREE is as follows: Ce > La > Nd > Pr > Sm. According to Elsner,^1^ there is an increase in LREE in monazites in the series grading from granite-pegmatites to granites to alkaline rocks and finally to carbonatites. Consequently, the studied monazite is probably derived from granites in alkaline rocks.

#### Rutile (TiO_2_)

Rutile occurs in forms are prismatic red and black, rounded, yellow, deep blood red, and black (Fig. S10a). The chevron (elbow) rutile variety is common. Rounded rutile grains indicate recycled sedimentary source rock.^50^ The main paramagnetic rutile grains were separated at a magnetic field strength of 1.5A, and the rest diamagnetic rutile was concentrated in nonmagnetic field strength of 1.5A. The opaque variety is frequent in the highly magnetic group, while yellowish and reddish (translucent) rutile is more numerous in the nonmagnetic group. Fe-Ti minerals such as magnetite, ilmenite, and rutile came from the volcanic rocks on the Ethiopian plateau, transported by the Nile River.^8^ In terms of TiO_2_%, the studied rutile is classified into two types: high-grade rutile with TiO_2_ ~ 95 wt% (Fig. S10c, d) and low-grade rutile with TiO_2_ ~ 88 wt % (Fig. S10b). In the studied area, the average rutile content is 0.086% in the beach samples and 0.060% in the dune samples.

#### Titanite (Sphene) CaTiSiO_5_

Titanite was recorded in all samples. Its color varies from yellowish to brownish-yellow with a vitreous luster (Fig. S11a). Titanite mineral grains are subhedral to anhedral with a sphenoid habit and are twined (Fig. S11 a, b, c). Titanite was separated into groups with magnetic field strengths of 1.0A and 0.5A (less common). EDX microanalysis data of the titanite grains are shown in Fig. S11b, c, displaying relatively high content in iron oxide: this causes a brownish color. In addition, no significant role was played by titanite in the radioactivity of the studied area. The mean content of titanite is 0.006% in the dune samples and 0.005% in the beach sand samples (Table 2).

### Accessory minerals

#### Thorite (ThSiO_4_)

**Figure. 8:**
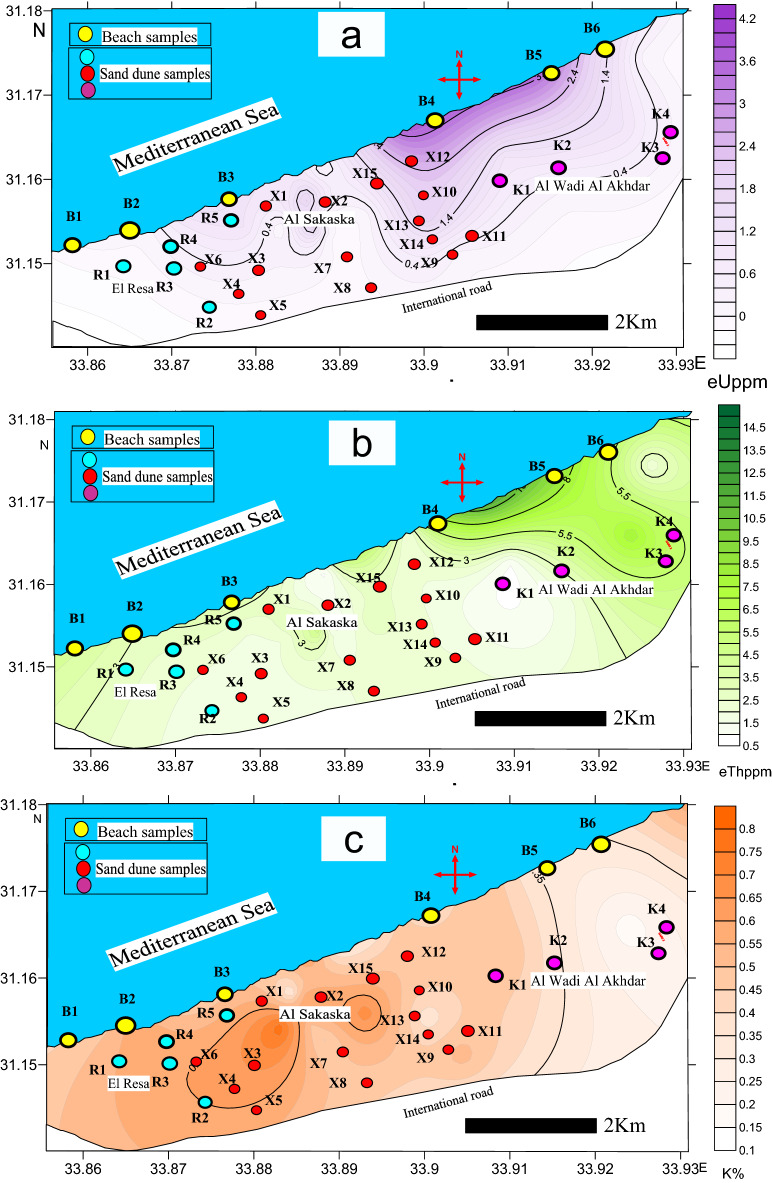
show: (**a**) eU distribution contour map, (**b**) eTh distribution contour map and (**c**) K % distribution contour map.Map was created by Surfer Software 13.6 (2016) https://www.goldensoftware.com/products/surfer.

Thorite is associated with xenotime, zircon, sphene, monazite, and allanite. Thorite’s color is mostly opaque brownish to reddish-brown (Fig. S12a): it has opaque red grains (Fig. S12c) and non-opaque reddish honey grains (Fig. S12e). EDX microanalysis data (Fig. S13b, d, f) shows that thorite grains contain a Th oxide content ranging from 62.3 to 66.54, averaging 64.02, while U oxide content ranges between 2.27 and 12.49%, averaging 6.79%. Moreover, opaque thorite (Fig. S12c, d) is more uraniferous (U oxide = 12.49 wt%) than non-opaque thorite (Fig. S12a, b, e, f). Additionally, the distribution of eTh in ppm in the study area (Fig. 8b) seems to be controlled by monazite and thorite.

#### Apatite (Ca_5_(PO_4_)_3_(F,Cl,OH))

Apatite in the studied sediments is relatively absent compared to other heavy minerals. The most frequent apatite grains are extensively rounded, egg-shaped, or spherical and exhibit various colors ranging from black and greyish-black (Fig. S13a) to dark brown (Fig. S13b), orange (Fig. S13c), dark yellow (Fig. S13d), light yellow (Fig. S13e), and colorless (Fig. S13f.). This diversity of colors is probably due to the presence of ferric or ferrous iron and other trace elements. From the results of this work, we can conclude that rounded apatite grains (Fig. S13) in the sedimentary record^50,63,64^ are likely to have undergone polycyclic reworking over several sedimentary cycles and during aeolian transport. EDX microanalysis (Fig. S13g–j) shows a relatively low U and Th value compared to other recorded radioactive minerals, such as monazite and thorite.

#### Xenotime (YPO_4_)

The major component of xenotime is yttrium orthophosphate (YPO4), which occurs as yellowish translucent brown grains (Fig. S14a). It is a soft mineral (Mohs hardness 4.5), with a specific density ranging from 4.4 to 5.1 g/cm^3^. Its luster, which may be vitreous to resinous, together with its crystal system, may lead to confusion with zircon. However, the cleavage (which is perfect in two prismatic directions) and softness of xenotime are sufficient to distinguish it. A few grains of xenotime were found amidst the heavy minerals. The EDX and BSE microanalyses are graphically represented in Fig. S14b.

#### Reserve estimation of the economic heavy minerals

The volume of raw sand in the studied area is calculated by multiplying the length, width, and depth of the sediments. The tonnage of the raw sand of these sediments is calculated by multiplying the volume of the raw sand by the specific density of the sediments, as shown in Table 7. The tonnage of total economic minerals was calculated by multiplying the tonnage of the raw sand (ton) by the average calculated weight percentage of the total economic minerals (Table 8). The tonnage of individual economic minerals was calculated by multiplying the tonnage of the raw sand per ton average and the calculated weight percentage of individual economic minerals divided by 100 (Table 9).

**Table 7:** The tonnage of the raw sand of the studied area.

	Volume (m^3^) Length × width × depth	Specific gravity of the raw sand (ton/m^3^)	Tonnage of the raw Sand (ton)
Sand dune	10,500 × 2500 × 25 = 656,250,000	1.56	1,023,750,000
Beach	10,500 × 4 × 6 = 252,000	1.58	398,160

**Table 8:** The tonnage of total economic minerals in the studied area.

	Tonnage of the raw Sand (ton)	The average weight percent of total economic heavy minerals	The average reserve of total economic minerals (ton)
Sand dune	1,023,750,000	1.915	19,604,812
Beach	398,160	3.943	15,699

**Table 9:** The tonnage of individual economic mineral in the studied area.

Economic heavy mineral	Sand dune		Beach	
	Economic heavy minerals %	The average reserve(ton)	Economic heavy minerals %	The average reserve(ton)
Magnetite	0.058	593,774.98	0.112	445.93
Ilmenite	1.341	13,728,487.15	2.526	10,057.23
Leucoxene	0.073	747,337.48	0.063	250.83
Rutile	0.060	614,249.98	0.086	342.41
Zircon	0.203	2,078,212.45	0.410	1,632.41
Monazite	0.007	71,662.50	0.024	95.56
Titanite	0.006	61,425.00	0.005	19.91
Garnet	0.167	1,709,662.46	0.716	2,850.74

### Radioactivity of the studied samples

The natural radioactivity in the studied sand dune and beach sand samples is based on the three main naturally occurring radioactive elements: U, Th, and K. Radiometric measurements for eU, eTh, and K were carried out using a Bicron-scintillation NaI (Tl) detector connected to a multichannel analyzer. The distribution values of eU (ppm), eTh (ppm), and K% were graphically plotted as contour maps (Fig. 8a, b, c, respectively).

The radioactivity data indicates that the beach sand samples are more radioactive than the sand dune samples, especially in the eastern direction, as represented by samples 4 and 5. The eU values (Fig. 8a) and eTh values (Fig. 8b) of beach sand reach 4 and 15 ppm, respectively. K content was almost uniformly distributed throughout the study area, as shown in Fig. 8c.

1. The mineralogical investigation of the studied area explains the generally marked enrichment of total heavy minerals in beach sand, with an exceptional increase of THM in samples B4 and B5 (Figs. 6, 7 and Table 1). Thus, the beach sand samples have higher levels of monazite, zircon, and thorite, which are considered the main source of radioactivity in the studied area.
2. ESEM data revealed that:
3. Zircon grains (Fig. S5 and Table 5) have Th oxide content varying from nil to 3.96%, with a mean of 2.04%, while U oxide content ranges between nil and 2.50%, averaging 0.74%.
4. Monazite grains (Fig. S9) have Th oxide content varying from 10.14 to 12.67%, with a mean of 11.23%, while U oxide content ranges between 1.77 and 2.67%, averaging 2.15%.
5. Thorite grains (Fig. S12) have Th oxide content ranging from 62.3 to 66.54%, averaging 64.02%, while U oxide content ranges between 2.27 and 12.49%, averaging 6.79%.

Consequently, the radioactivity of the studied area is related to monazite and zircon rather than thorite. Monazite grains contain Th and U, which indicates that radioactivity is mainly related to thorium rather than uranium. Finally, the mineralogical evaluation data of THM, especially monazite and thorite, are compatible with the radioactivity data.

## Conclusion

Egyptian beach black sands and sand dune deposits are discontinuously distributed along the Mediterranean coast, including the El-Arish coastal area in north Sinai, Egypt. Several drill hole samples have been used to represent the main two geomorphologic units in the studied area: beach sand and sand dunes. These samples were homogeneously mixed and collected to give one composite sample for the individual wells. The separation of total heavy minerals using heavy liquid and electromagnetic techniques was conducted. The study focuses on minerals with the highest economic value (total economic heavy minerals, TEHM), such as ilmenite, leucoxene, magnetite, garnet, zircon, rutile, monazite, and titanite. Those minerals with low economic value, namely green silicates (amphiboles, pyroxene, mica, and others), were discarded. The natural physical concentration of the Mediterranean current is responsible for the high concentration of THM in the beach sand. The mean THM of beach sand is 4.5% and 2.61% for sand dunes. For all samples, opaque minerals, especially ilmenite, magnetite, and leucoxene, made up to ~ 70% of the economic heavy mineral content. There is wider variation in the non-opaque minerals, dominated by garnet, zircon, rutile, apatite, and titanite monazite, among others. Mineralogical examinations were performed with ESEM, identifying more radioactive minerals, such as monazite and zircon. The ESEM analyses of monazite indicate that the Th and U content, averaging at 11.24% and 2.17%, are the main minerals responsible for radioactivity in the studied area. Additionally, no significant role was played by opaque minerals in the radioactivity. We have summarized the use of studying heavy minerals and have provided provenance interpretations. The total reserve per ton for each economic heavy mineral was calculated. The highest value is related to ilmenite: 13,728,487.15 tons of sand dunes and 10,057.23 tons of beach sand. The lowest value is related to titanite: 61,425 tons of sand dunes and 19.91 tons of beach sand.

## Supplementary Information

Supplementary Information.

## Data Availability

The authors declare that all data supporting the findings of this study are available within the paper and the Supplementary Information.
