# Supplementary material for: Separation, reserve estimation and radioactivity responsibility of the economic heavy minerals of East El- Arish black sand, North Sinai, Egypt
Source: Sci Rep. 2023 Mar 21;13:4608. doi: 10.1038/s41598-023-31440-y (PMC10030898; doi:10.1038/s41598-023-31440-y)
Supplement: Supplementary file 1 — Supplementary Information. [file 41598_2023_31440_MOESM1_ESM.docx]

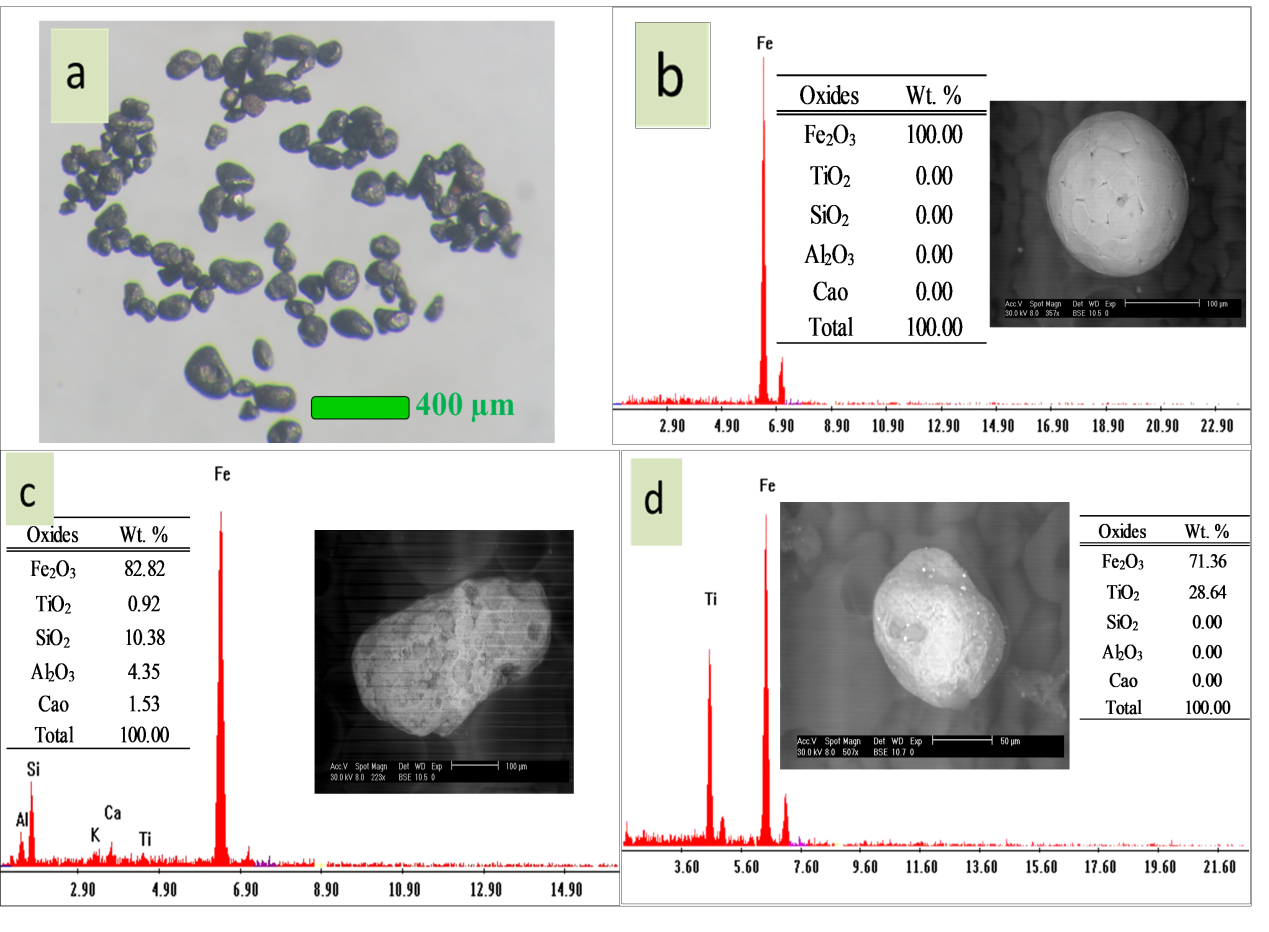


Figure.**S**1. Photomicrographs show: (a) massive, granular, angular to sub- angular, (b and c) EDX and BSE image of magnetite grain and (d) EDX and BSE image of titano-magnetite grain.


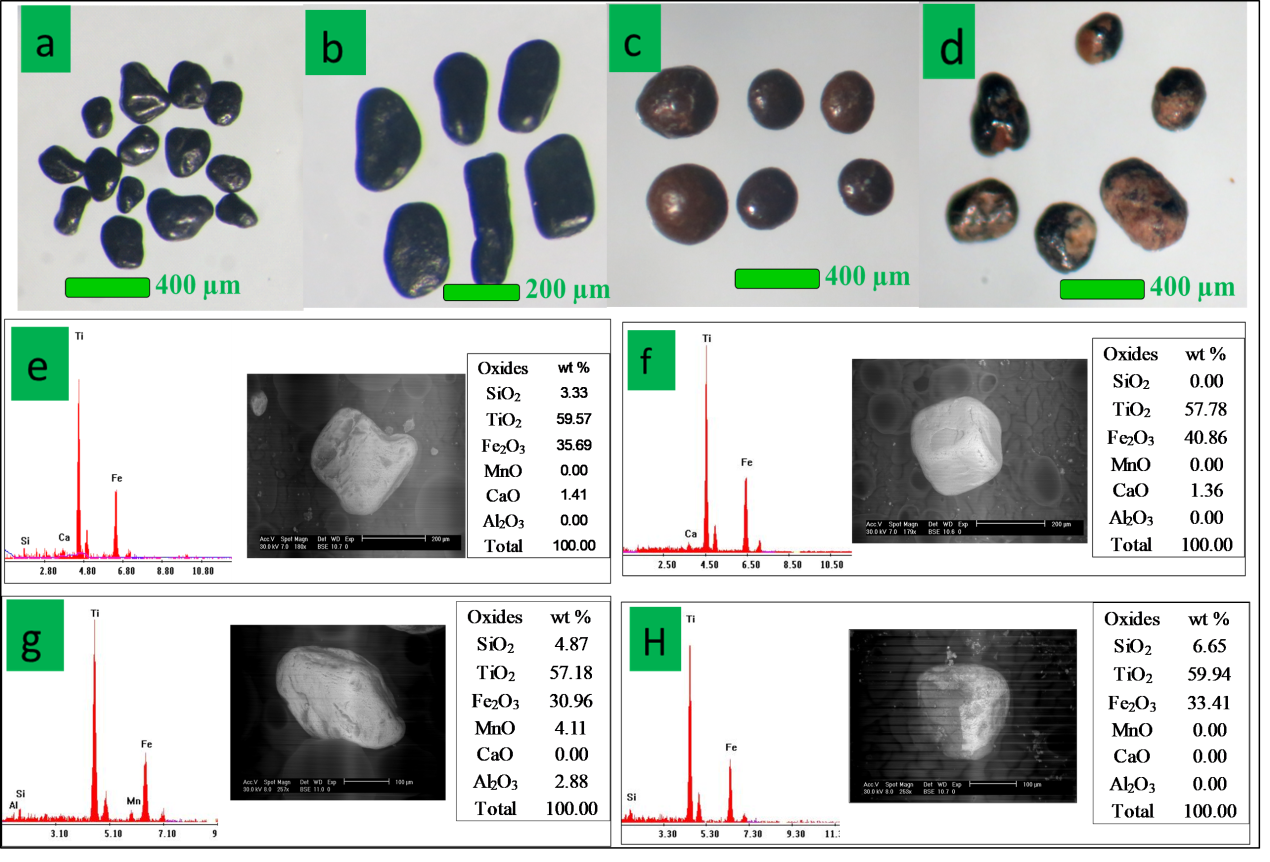


Figure. **S**2. Photomicrograph shows different ilmenite forms: a) irregular angular to subangular with smooth edges, b) rod like shape, c)oval with smooth and pitted surface and well rounded to subrounded and (d, e, f &g) EDX and BSE image of ilmenite grains.


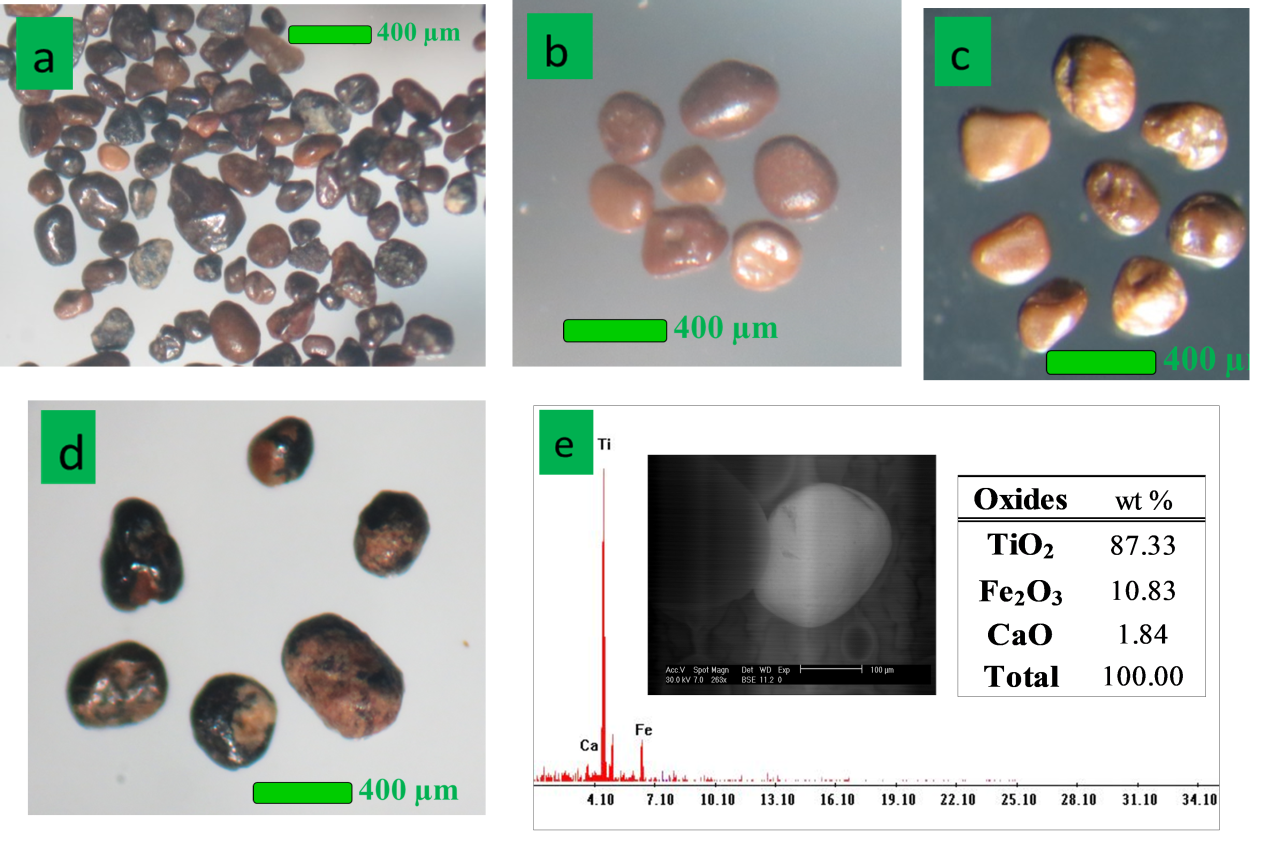


Figure. **S**3. Photomicrographs show: a) varieties of leucoxene, b) Sub sounded brown leucoxene, c) light brown leucoxene d) grains explain the alteration process of ilmenite to leucoxene e) EDX and BSE image of leucoxene grains.


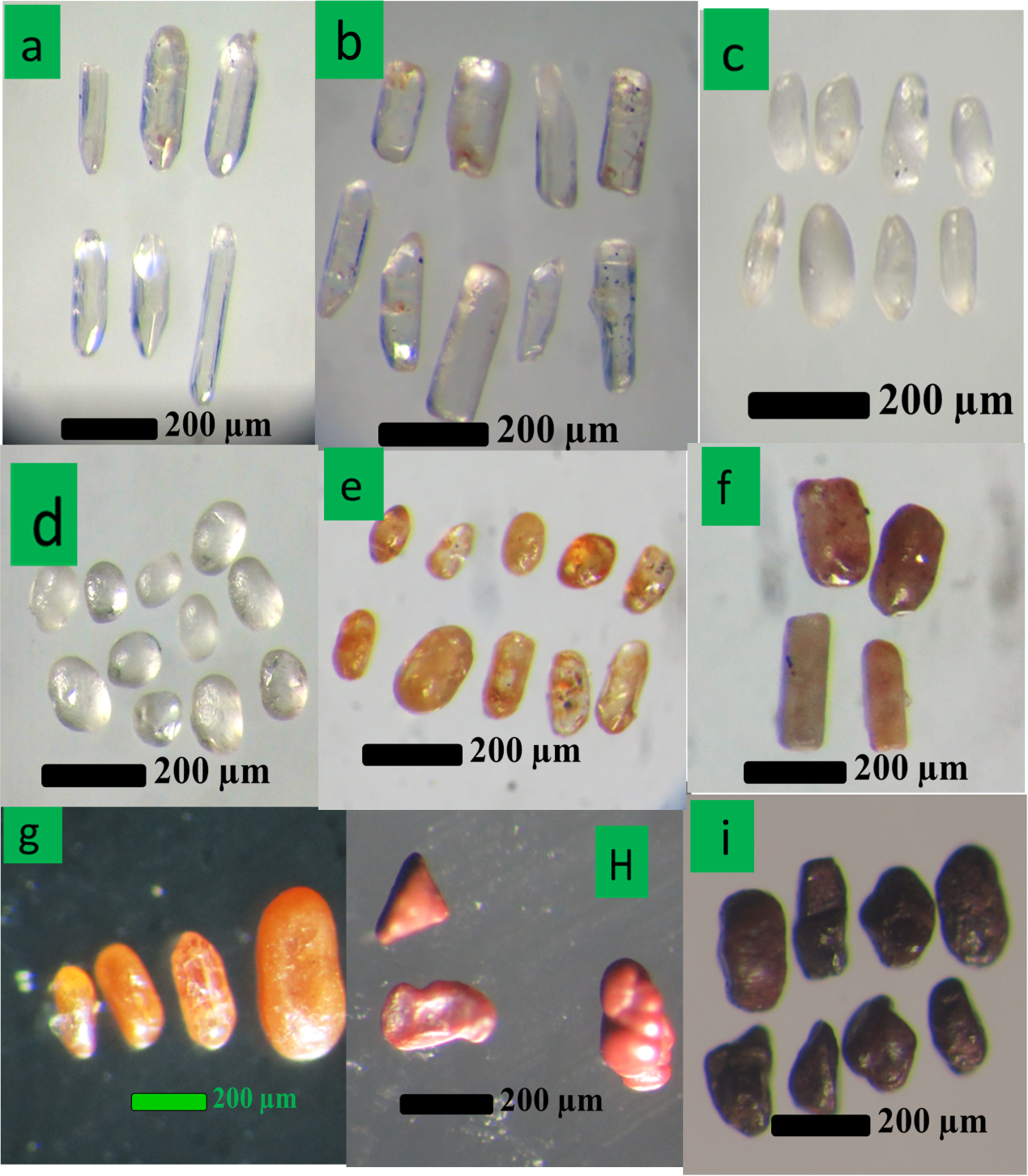


Figure. **S** 4. Photomicrographs showing the diversity of zircon varieties: a) colorless short to long prismatic with bipyramid termination (water clear zircon), b) colorless prismatic with black inclusions, c) colorless oval shape, d) colorless rounded shape, e) yellow prismatic, f) muddy color, g) orange, h) red euhedral and i) black grains.


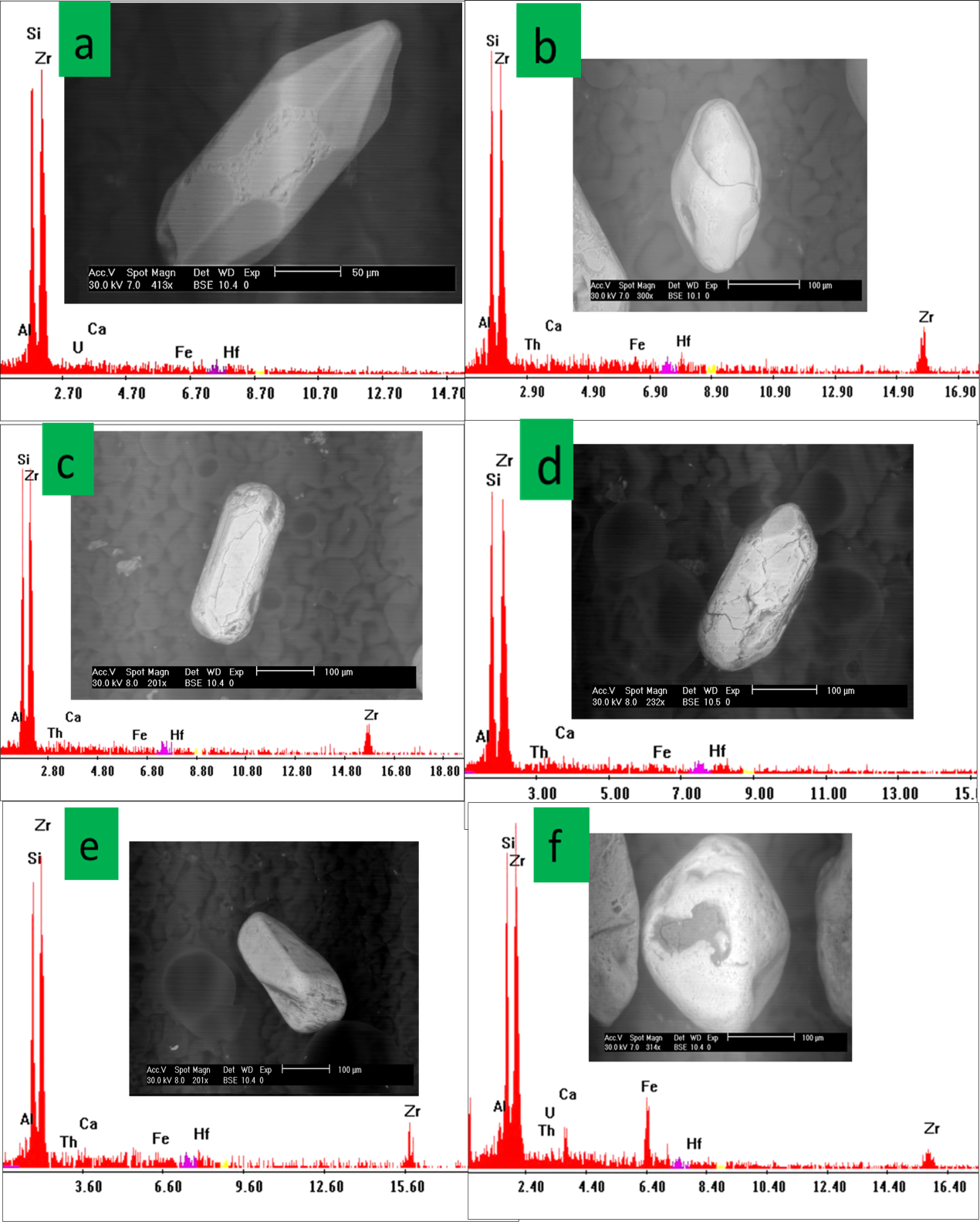


Figure. **S**5: EDX and BSE images of pure zircon grains: a) typical euhedral zircon crystal, b) bipyramid zircon grain, c and d) fractured prismatic zircon grains, e) irregular zircon fragments and f) bipyramid)


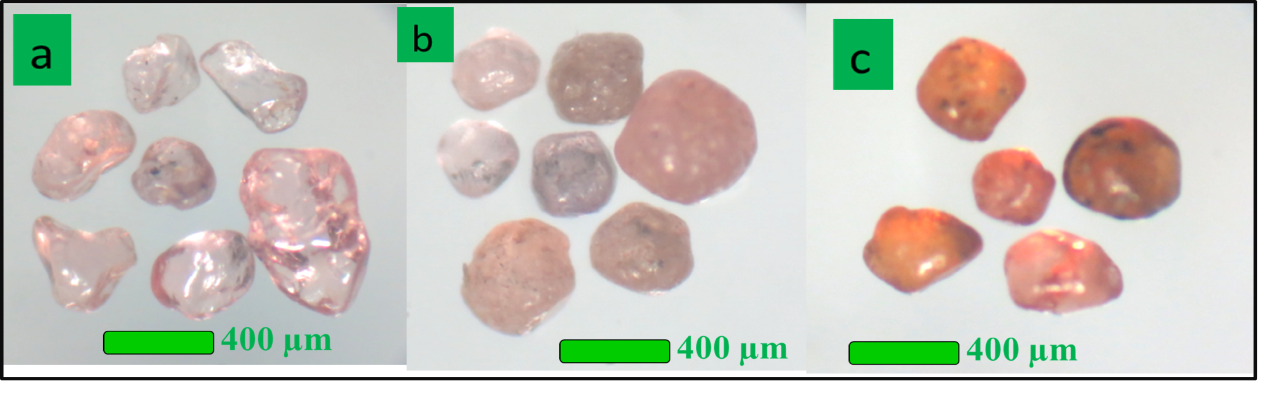


Figure. **S**6. Photomicrographs show: a) platy rose garnet grains, b) rose garnet grains with black inclusions and c) red garnet.


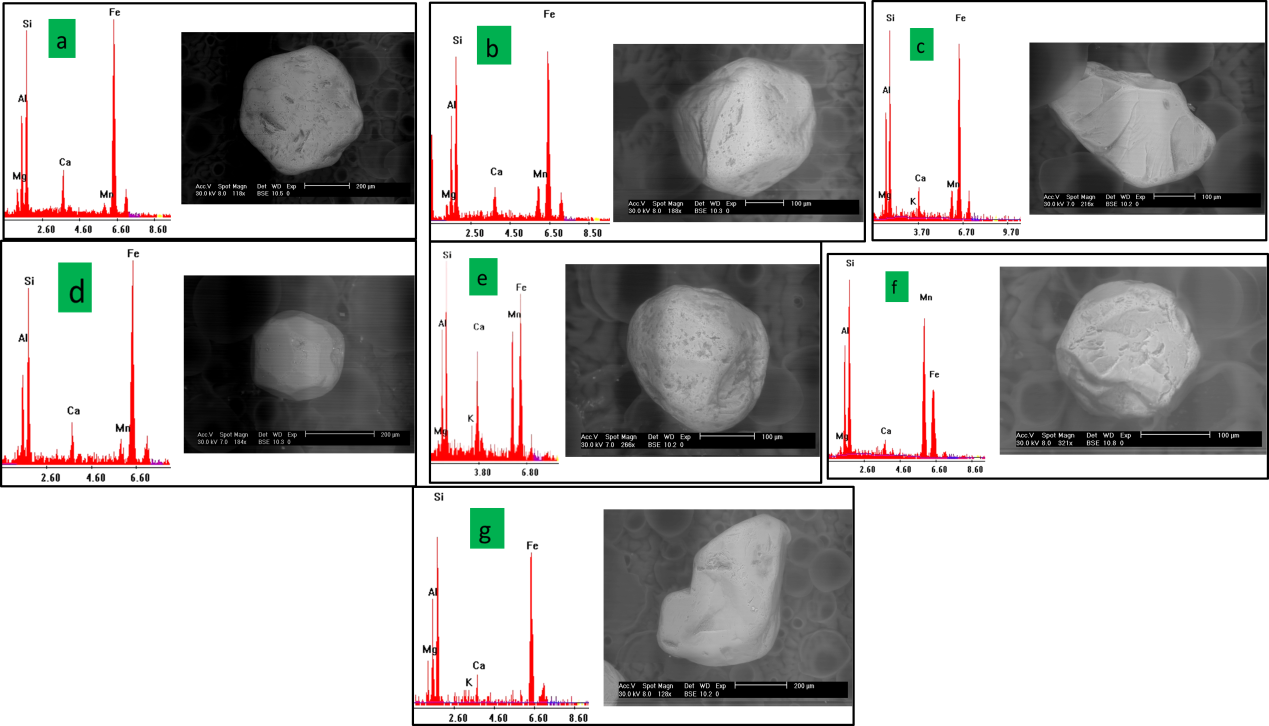


Figure. **S**7. EDX and BSE of garnet shows: a, b, d, g ) pitted surface, rounded edges of almandine garnet crystals c, g) irregular crystal of almandine garnet e &f) EDX and BSE image of almandine-spessartine solid solutions.

Figure. **S**8 . Subdivision of seven garnet grains Fe+Mn-Mg-Ca ternary plot showing definitions of garnet types A, Bi, Bii, Ci, Cii and D among the studied area after Mange and Morton (2007).Type A—granulite-facies metasediments and intermediate felsic igneous rocks (high Mg, low Ca), Type Bi-intermediate to felsic igneous rocks (high Fe, high Mn), Type Bii-medium-low metasedimentary rocks, amphibolite-facies (low Mg, variable Ca), Ci-metabasic rocks, Type Cii-ultramafic rocks, Type D-low-grade metabasic rocks or contact metasomatic metamorphic rock (Ca-rich).


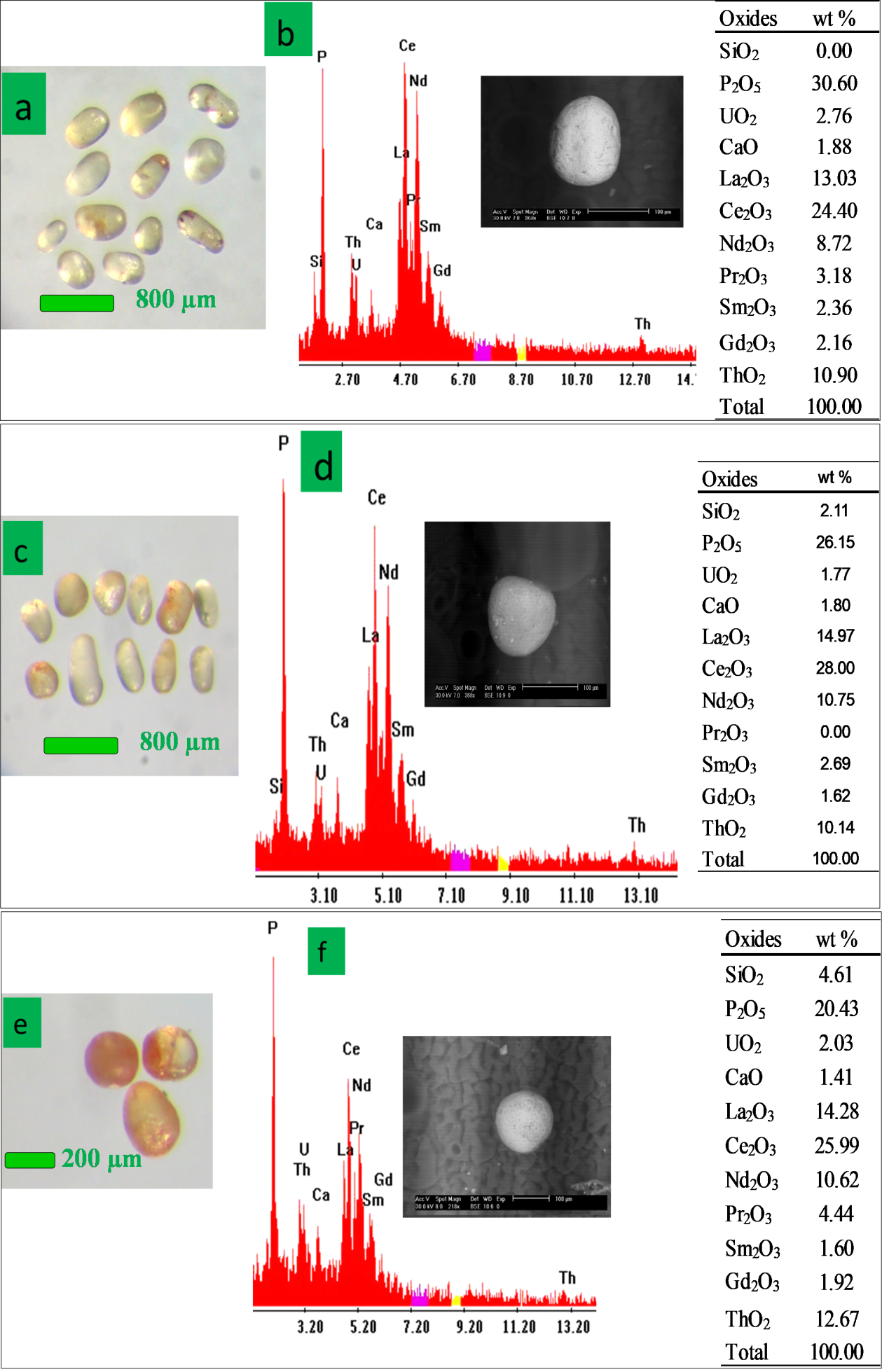


Figure. **S**9: Photomicrographs show: a) colorless monazite grains, b) EDX and BSE image of picked colorless monazite, c) lemon yellow monazite grains, d) EDX and BSE image of picked lemon yellow monazite, e) red monazite grains and c) EDX and BSE image of picked red monazite.


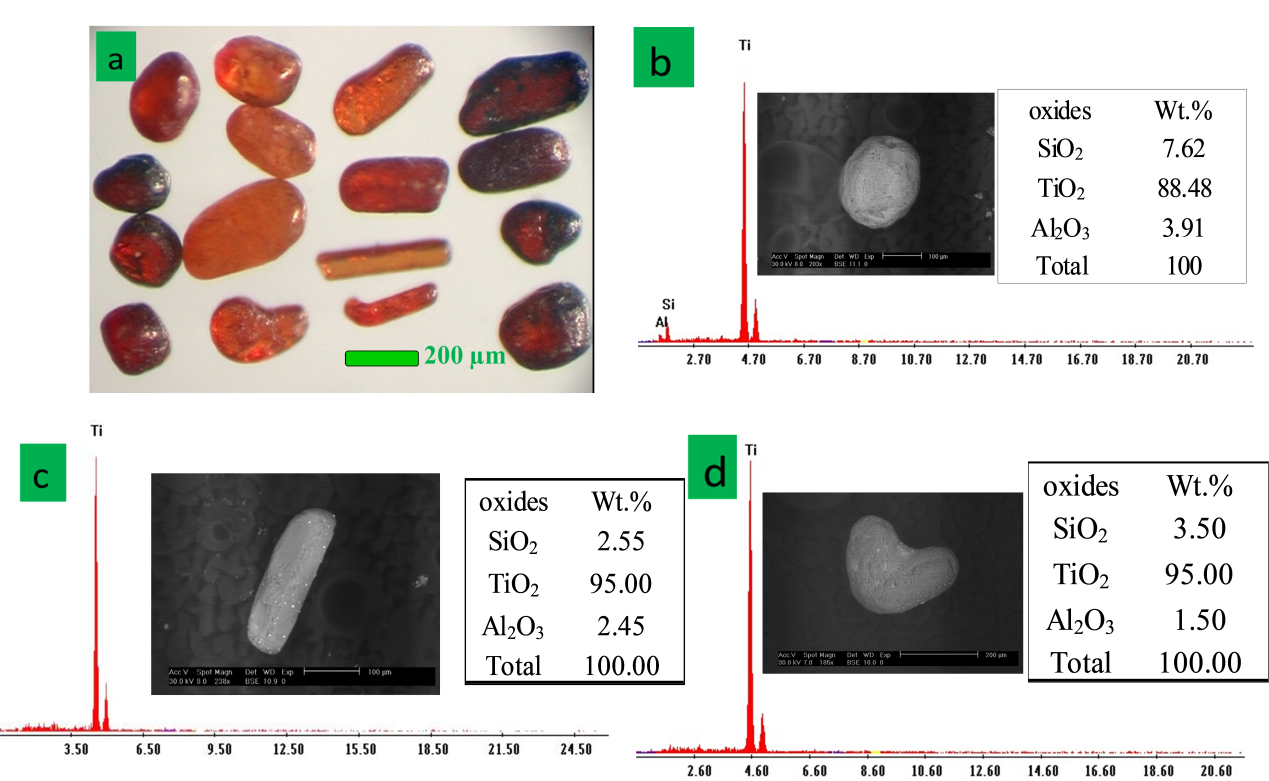


Figure. **S**10.Photomicrographs show: prismatic red and black rutile, rounded, yellow, red and black rutile, rounded and b, c &d) EDX and BSE image of rutile grains.


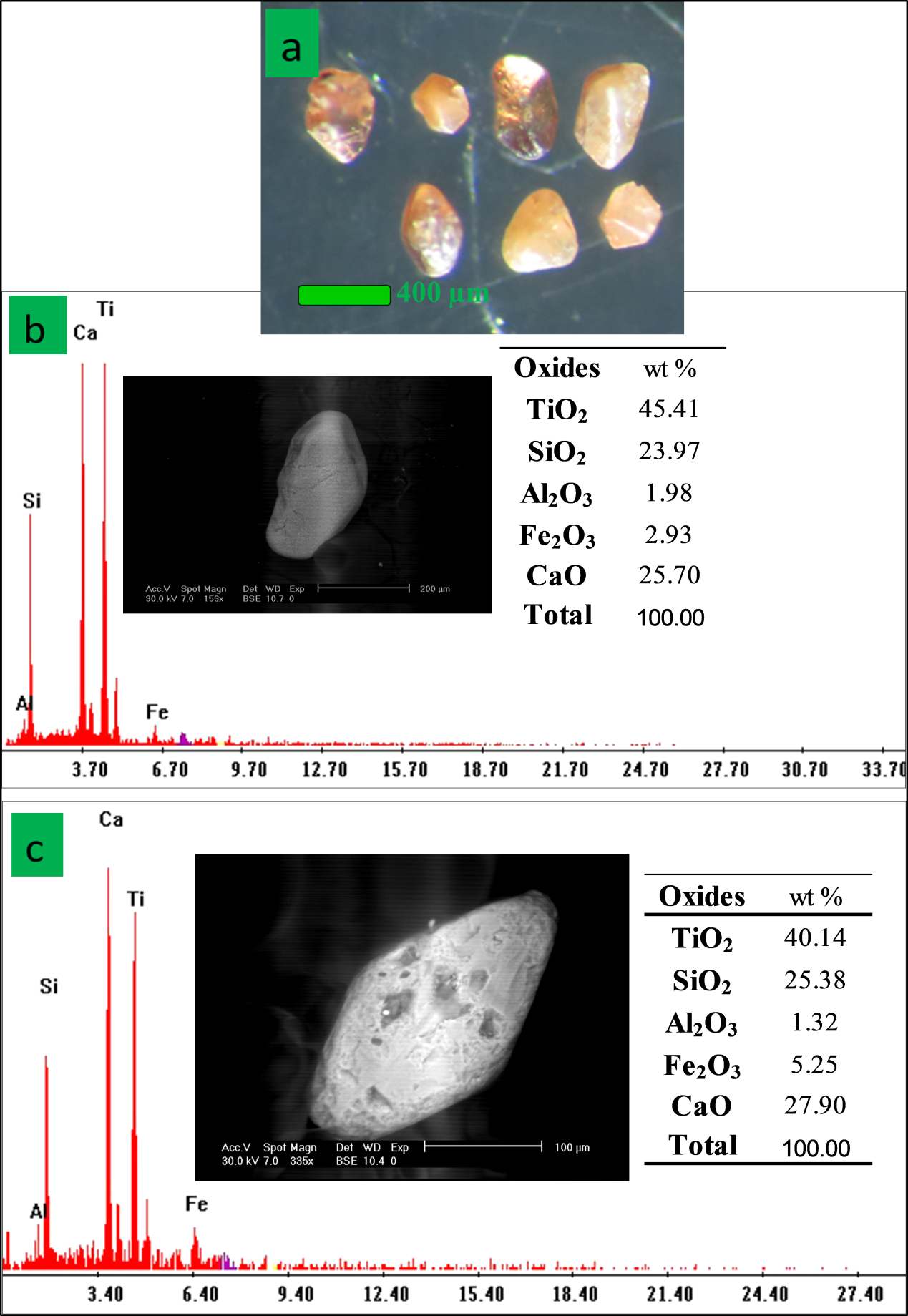


Figure. **S**11: Photomicrographs show: a) yellowish to brownish yellow titanite grains, b,c) EDX and BSE image of pure titanite grain.


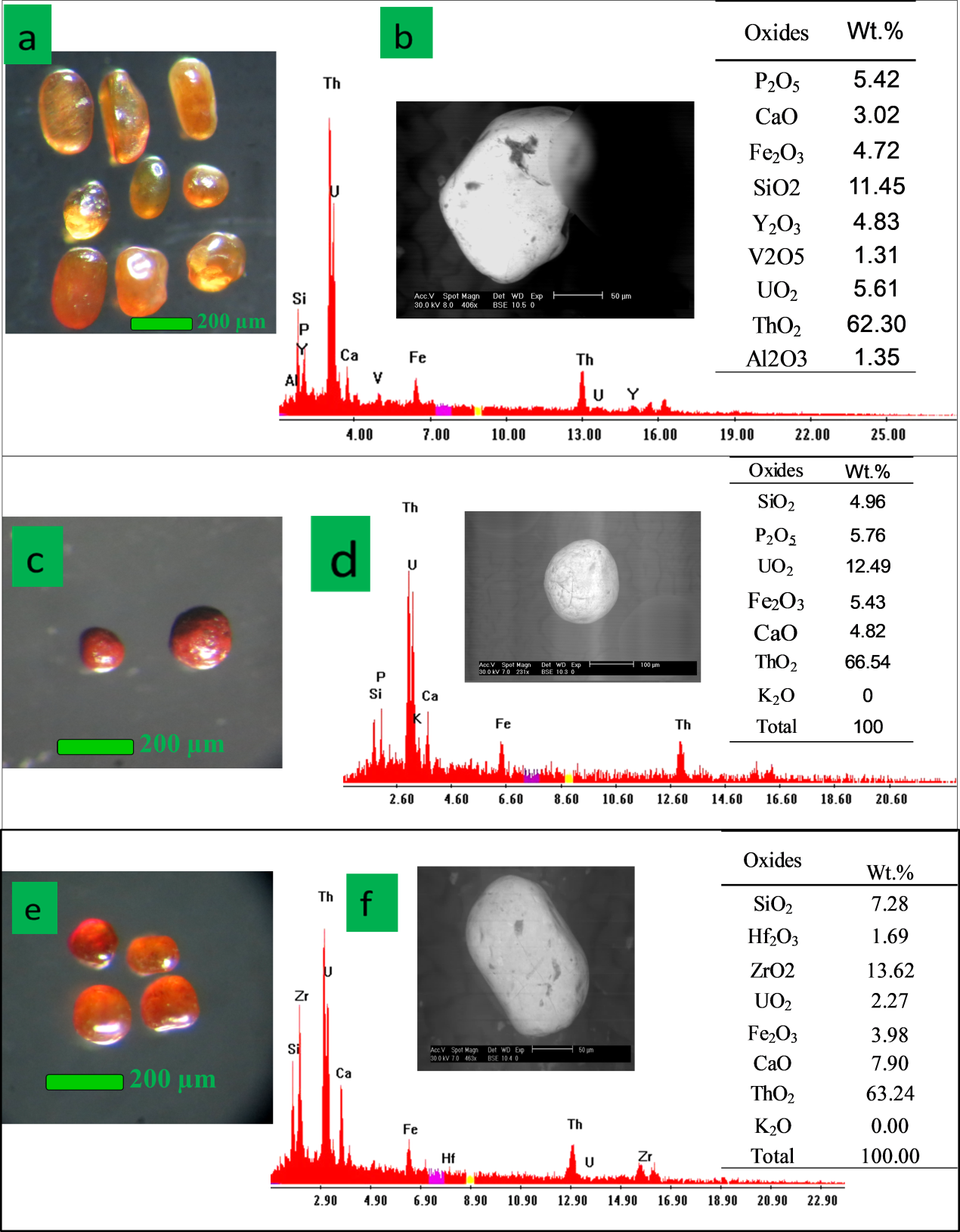
 Figure. **S**12. Photomicrographs show different thorite grains: a) opaque brown to reddish brown, b) non- opaque red grains, c) non-opaque reddish yellow and d, e &f) EDX and BSE image of pure thorite grains.


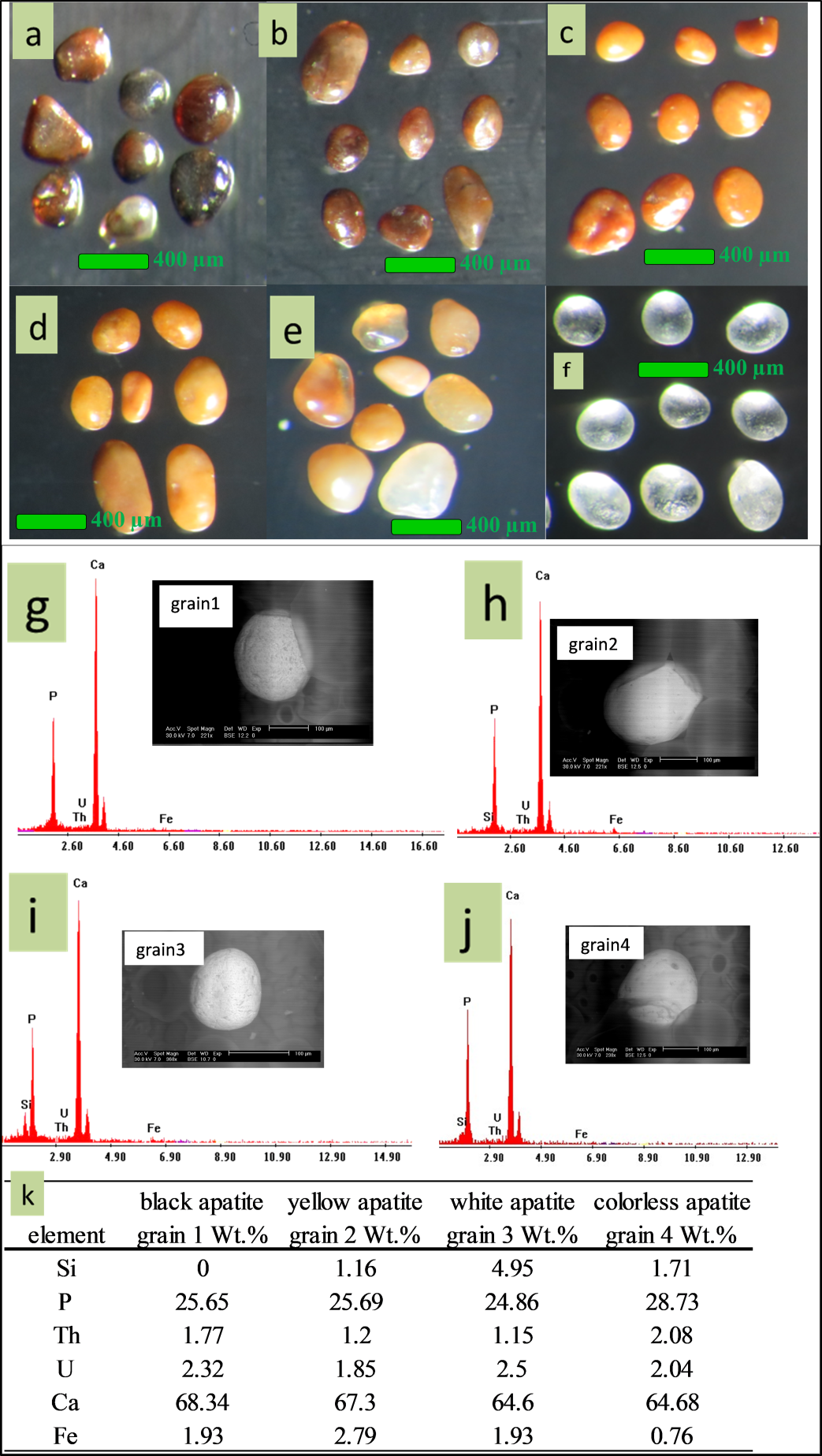


Figure.**S**13.Photomicrographs of apatite shows: a) black, b) dark brown, c) orange, d) dark yellow, e) light yellow, f) colorless and g, h, I &j) EDX and BSE image of pure apatite grains.


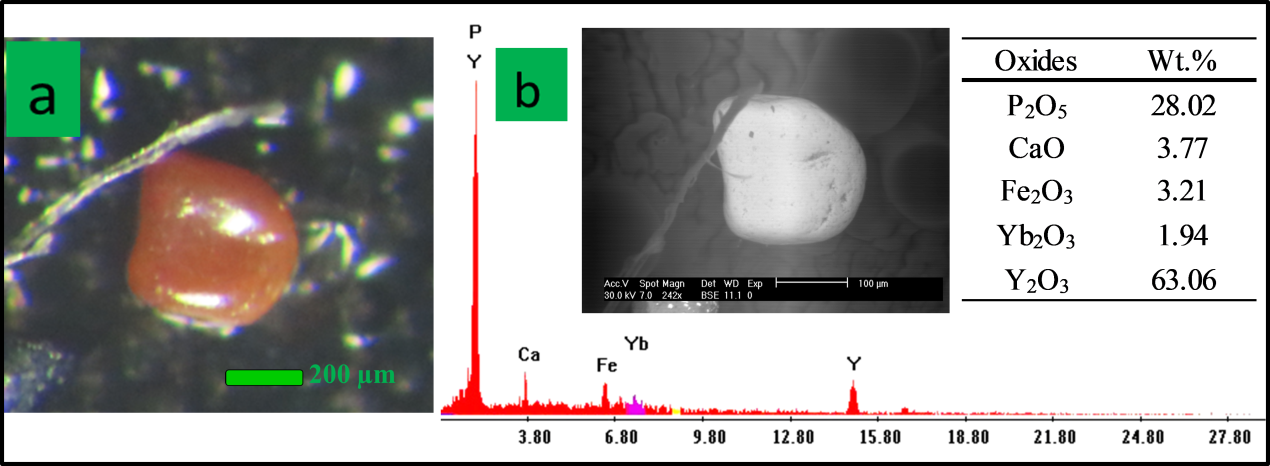


Figure. **S**14.Photomicrographs shows: a) brown xenotime and b) EDX and BSE image of pure xenotime grains.
